# The Influence of the ‘Trier Social Stress Test’ on Free Throw Performance in Basketball: An Interdisciplinary Study

**DOI:** 10.1371/journal.pone.0157215

**Published:** 2016-06-16

**Authors:** Nicolas Mascret, Jorge Ibáñez-Gijón, Vincent Bréjard, Martinus Buekers, Rémy Casanova, Tanguy Marqueste, Gilles Montagne, Guillaume Rao, Yannick Roux, François Cury

**Affiliations:** 1 Aix Marseille Univ, CNRS, ISM, Inst Movement Sci, Marseille, France; 2 Aix Marseille Université, LPCLS EA 3278, 13621, Aix-en-Provence, France; 3 Department of Kinesiology, KU Leuven, Tervuursevest 101, 3001, Leuven, Heverlee, Belgium; 4 Université de Toulon, Toulon, France; IRCCS Istituto Auxologico Italiano, ITALY

## Abstract

The aim of the present study was to explore the relationship between stress and sport performance in a controlled setting. The experimental protocol used to induce stress in a basketball free throw was the Trier Social Stress Test (TSST) and its control condition (Placebo-TSST). Participants (n = 19), novice basketball players but trained sportspersons, were exposed to two counterbalanced conditions in a crossover design. They were equipped with sensors to measure movement execution, while salivary cortisol and psychological state were also measured. The task consisted of two sequences of 40 free throws, one before either the TSST or Placebo-TSST and one after. Physiological and psychological measures evidenced that the TSST induced significant stress responses, whereas the Placebo-TSST did not. Shooting performance remained stable after the TSST but decreased after the Placebo-TSST. We found no effect of the TSST or Placebo-TSST on movement execution. A multivariate model of free throw performance demonstrated that timing, smoothness and explosiveness of the movements are more relevant to account for beginner’s behavior than stress-related physiological and psychological states. We conclude that the TSST is a suitable protocol to induce stress responses in sport context, even though the effects on beginners’ free throw performance and execution are small and complex.

## Introduction

Stress is an integral part of the competitive settings that may be found in sport contexts [1]. Actually it has been documented that stress may affect sport performance and have direct or indirect influences on the outcome [2]. Note that the term stress refers to both the perception of uncontrollable and unpredictable situations and the set of psychological, behavioral and physiological responses triggered by these perceptions [3]. Combined, stress perceptions and stress responses constitute a system for anticipatory adaptation to perceived environmental challenges. In this sense, stress-related adaptations extend the temporal scope of homeostatic regulation by virtue of anticipatory processes that involve perceptual, motor, cognitive and neuro-hormonal interactions [4].

The magnitude and quality of stress responses can be estimated with physiological, psychological and behavioral methods. The neuroendocrine response is particularly adapted to stress research in general and to sport competition in particular [5]. For example, salivary cortisol is recommended for sports contexts [6] not only because it is a useful marker of the stress response [7] but also since a rise in cortisol secretion has been generally found in response to a competition [8]. In addition, this method is noninvasive, easy to collect, ethically inoffensive and pain free compared to collecting a blood sample [9]. Self-reported measures are also used to attest psychological stress responses, such as State and Trait Anxiety Inventory (STAI) [10] or Self-Assessment Manikin (SAM) [11]. The coordination, stability and flexibility in the control of postures and movements have also been used to estimate stress responses [12,13], although most studies had focused on performance as a final outcome without concentrating on the execution and control of movements.

Studies about the relationships between stress responses and sport performance have led to mixed results. For example, no relationship was found between cortisol level and performance for dancers [14], while a negative relationship was observed for golfers in a 36-hole golf competition [15] and a positive relationship was evidenced for weight-lifting [16]. Similarly, the impact of self-reported anxiety upon sport performance remains unclear [17]. From a motor control perspective, a reduced joint range of motion was found in a batting task [18] and the amplitude of arm and club movements decreased in a golf putting task [19], whereas no significant difference in movement kinematics was found during the execution of a table-tennis shot when exposed to either low or high anxiety conditions [13]. In the cognitive domain, the relationship between stress and performance is very often described as an inverted U-shape law [20]. According to this characterization, cognitive performance achieved under intermediate levels of stress is optimal and decreases when stress levels are too high or too low. However, in the domain of sport sciences, the debate regarding the existence of optimal levels of stress for performance is still intense [21], because sport skills elicit complex stress responses with qualitatively different mixtures of physiological and psychological components [9,22]. Therefore, it is still unclear what the optimal cortisol levels are, even though it has been shown that in general the relationship between cortisol levels and sport performance cannot be characterized as the present time by an inverted U-shape [21]. The contradictory findings about the relationship between stress and performance in sport contexts are often explained by a variety of interacting factors, as there are, the specificities of each sport (e.g., “stereotyped” movement in a golf putting task, vs., the visual information gathering by players during a table-tennis shot, or the high muscular strength in weight-lifting), the complexity of the stress responses themselves or the difficulty to isolate in some sports physiological from psychological stress. In addition, trying to manipulate the stress levels in an official game would certainly be rejected by both players and coaches. To further investigate these issues, the present study used a controlled setting (the Trier Social Stress Test, TSST) and a specific sport skill (basketball free throw).

The TSST [23] is one of the most popular and standardized methods to induce acute psychosocial stress in experimental settings [7], as the socio-evaluative character and the uncontrollability of the TSST create a robust stress response [24]. The TSST also affects multiple biomarkers of stress (for a review, see [7]), especially an important increase of salivary cortisol. Compared to the Socially Evaluative Cold Pressor Task (SECPT) and a computerized Mental Arithmetic Task (MAT), the TSST is the method that creates the strongest effects on mood and physiological measures, and generates the longest duration of the stress hormonal response (10–20 minutes after the stress inducing manipulation ended), long enough to perform an experimental task [25]. Despite the large body of literature that inspects the relationships between TSST-induced stress and cognitive performance, up to date only three studies used the standardized TSST in the sport context (an additional study [26] only focused on the second part of the TSST), while none of them used the specific control condition called Placebo-TSST [27]. These three studies evidenced that physically exercising young women [28] and elite sportsmen [29,30] had reduced but significant physiological and psychological responses to TSST-induced stress compared with untrained women or men. Even though these three studies provide interesting general findings, concluding results on the effects of the TSST on sport performance for specific tasks are still missing. To that aim, the present study focuses on basketball free throw shooting.

Free throw shooting was selected for several reasons. (1) The free throw can be considered as a standardized performance task because the shooter has full control of his “stereotyped” movement, other players are not allowed to interfere and the environment is not variable. (2) The rise in cortisol following psychosocial stress can be more easily isolated because the effects of physical exercise on cortisol levels can be neglected in a sequence of free throws [31]. (3) Free throw shooting is one of the most important and anxiety provoking game situations in basketball [32]. (4) Stress impacts the gross performance, but also the motor preparation and organization of the shot (even for elite players), as evidenced by longer preparation times and more frequent overthrown shots [33]. In sum, free throw is an appropriate task to examine in detail the impact of the TSST on sport performance and execution.

The aim of this work is two-fold. First, to assess the suitability of the TSST as a method to study the effect of stress on performance in realistic sport contexts. Second, to analyze the execution and performance of basketball free throwing in neutral and psychosocially stressful contexts. We used an interdisciplinary methodology to better understand how physiological, psychological and behavioral stress responses affect performance in basketball free throwing [34]. While we predicted that the TSST would induce a rise in cortisol and an increased self-reported anxiety, specific predictions about the second aim are less straightforward, as positive and negative influences have been found previously.

## Materials and Methods

### Participants

The study was approved by the institutional board of the University. Prior to data collection, all participants signed an informed consent following requirements of the Declaration of Helsinki. They were informed of their right to stop their participation at any time. Nineteen men (20.66 ± 1.94 years with a range of 18–25 years; Body Mass Index of 22.18 ± 2.07 kg/m^2^) voluntarily participated in the study. They were all competitive sportsmen (7.61 ± 3.10 hours of sport per week), yet still novices in basketball. The selected participants were not elite sportsmen in their respective sports because experts present significantly lower cortisol responses to TSST than untrained men [29]. Women were excluded from the study because oral contraceptives and menstrual cycle could have influenced cortisol levels [35]. Additional exclusion criteria were: smoking more than five cigarettes a day, drinking more than two glasses of alcohol a day, medication intake, drugs abuse, reported medical illness, history of endocrine disorder, increased levels of chronic stress (French version of the Perceived Stress Scale, PSS [36]), cardiovascular diseases, and psychological distress (French version of the General Health Questionnaire, GHQ-12 [37]).

### The Trier Social Stress Test (TSTT)

The standardized TSST is an exposure to a socio-evaluative stressful situation, with a speech and a math test in front of two unknown experimenters and a video camera. Participants were told that their performance would be videotaped for further analysis, because the experimenters were specialized in observing nonverbal behavior. A control condition (Placebo-TSST) was designed to be analogous to the TSST without being stressful for the participants [27]. After a 3-min preparation period, participants were instructed to read a text aloud during five minutes alone in a room, and then they were asked to complete a simple arithmetic task for five minutes. Participants were standing during the two tasks to be as close as possible to the TSST condition before the second sequence of free throws following the Placebo-TSST.

### Experimental Design and Procedure

**Fig 1:**
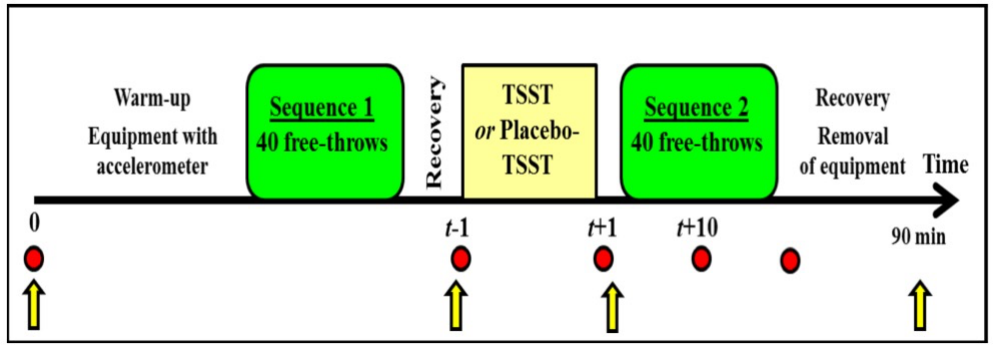
Timing of saliva sampling, completion of questionnaires and free throws performance represented the study design. Notes: *t* is the time in minutes when the cortisol level was assessed according to the temporal location of the TSST or the Placebo-TSST; red circles are the moments of saliva collection; yellow arrows are the moments of the completion of questionnaires (STAI and SAM).

The experiment used a crossover design, often more powerful than a two-group comparison study and validated with the TSST and the Placebo-TSST [27]. Participants were invited in the laboratory twice. While they were confronted with the TSST or the Placebo-TSST condition in a first session, the opposite treatment was administered during a second session, at least 14 days after the first. The assignment of treatment order was randomized. Experimental sessions were conducted between 10:30 and 17:00 h and took about 90 minutes. Participants were welcomed followed by a short static warm-up session of ten free throws. After being equipped with the required sensors the participants were positioned on a force platform and performed a pre-test sequence of 40 free throws, followed by a recovery period of ten minutes before the beginning of the TSST or the Placebo-TSST that took place in another room. The tests began 40 minutes after the arrival of the participant to minimize effects of possible prior stressful events. Subsequently, a post-test sequence of 40 free throws was performed. After a recovery period, the equipment was removed, a last set of questionnaires was completed and participants were thanked for their cooperation. They were completely debriefed only after their second treatment. The full procedure is presented in Fig 1.

### Physiological, psychological and behavioral testing

#### Cortisol sampling and assays

Participants were instructed not to practice sport, not to use alcohol and not to take medication during the twelve hours preceding the experiment and not to eat or drink anything except water, not to brush their teeth and not to smoke one hour before the experiment. Increase of salivary cortisol after TSST exposure has been demonstrated in the morning and in the afternoon with equal reliability [35], so experimental sessions could be conducted between 10:30 and 17:00 h. Participants tested in the morning were asked to wake up at least three hours before the experiment to avoid the cortisol awakening response during the course of the experiment [7]. To control for the circadian rhythm of HPA axis activity, the second experimental condition (TSST or Placebo-TSST) took place during the same time slot as the first experimental condition. Saliva was collected five times via plastic saliva collection tube at baseline (-40 min before the TSST or Placebo-TSST treatment), just before the treatment (-1 min), just after the treatment (+1 min), in the middle of the post-test free throw sequence (+10 min) and at the end of the post-test free throw sequence (+15 min). Samples were immediately stored at -18°C, before being analyzed. Salivary cortisol levels were determined using enzyme-linked immunosorbent assay (ELISA) kits (Expanded Range High Sensitivity Salivary Cortisol Enzyme Immunoassay Kit—N° No. 1–3002, Salimetrics, UK). Absorbance at 450nm was measured with a 96 well-microplate spectrophotometer (Multiska FC Microplate Photometer, Thermo Fisher, Germany). Each well containing samples or cortisol standards and controls was duplicated. The intra-assay precision was determined from the mean of replicates with a single microplate (coefficient of variation 4.6±1.5%). The inter-assay precision was determined from the mean of average duplicates based on the cortisol standards wells for calibration (3.0, 1.0, 0.333, 0.111, 0.037, 0.012 μg/dL) for calibration, used in separate runs (coefficient of variation 6.0±3.7%), but prepared from the same stock solution and dilutions. The concentrations of unknown samples were then computed by interpolation using a 4-parameter non-linear regression curve fit, as recommended by Salimetrics’ protocol.

#### Self-reported measures

State anxiety was repeatedly measured with the French version of the State-Trait Anxiety Inventory (STAI) [10]. Participants answered to the twenty items on a four-point Likert scale (1 = not at all, 4 = very much so), after which all items were summarized into one composite variable. Furthermore, emotional state was assessed using the Self-Assessment Manikin (SAM) [11], a non-verbal pictorial assessment technique that directly measures the pleasure, arousal and dominance associated in response to an object or event. Participants circled a figure corresponding to their emotional state: from a smiling figure to a frowning figure for the pleasure dimension, from a large figure (excited) to a small figure (relaxed) for the arousal dimension and from a large figure (maximum control in the situation) to a small figure (lack of control) to assess the dominance dimension. These two self-reported measures were assessed at baseline (-40 min before the TSST or Placebo-TSST treatment), just before the treatment (-2 min), just after the treatment (+2 min) and at the end of the experiment (+25 min).

### Free throw performance measurement

Participants performed two sequences of 40 free throws, one before the treatment (TSST or Placebo-TSST) and one after. Visual inspection was used to determine free throw scoring and the series of shots were manually documented. Performance in the task was defined in two complementary ways. For the ANOVA we defined performance as the number of successful free throws in the pre-test and post-test blocks. For the logistic model of scoring probability, we defined performance as the probability of scoring a shot.

#### Movement execution measurement

Participants were equipped with 3DOF accelerometers (±2 g, Trigno, Delsys ®, Inc., Natick, MA) attached to the shooting arm (over the hand, forearm and upper arm segments) and along the spine (at the level of C7 vertebra and sacrum). These accelerometers provided measures of the 3D linear accelerations at 148 Hz. Ground reaction forces and moments were measured using a 600x400mm force plate (Kistler 9281 CA). The force-platform acquisition frequency was 1000Hz with an ADwin-Gold ® system, connected via Ethernet to a computer. The signals from the accelerometers and the force plate were synchronized with a Delsys Trigger Module. Raw signals from the accelerometers and the force plate were filtered with a 4-th order low-pass Butterworth filter with a cut-off frequency of 10Hz. The signals were time-aligned at the maximal ground reaction force (slightly before ball release) with a selected period of interest of ±0.5 s around this event.

Peak velocity and total jerk were computed from the acceleration data to characterize the kinematic profile of the body segments. We used a dimensionless normalization [38] to estimate jerk:

$$J=\frac{\Delta t}{v_{mean}^{2}}\int_{t1}^{t2}\dddot{x}{\left(t\right)}^{2}dt$$ (1)

where *Δt* is the time difference between *t*_*1*_ and *t*_*2*_, *v*_*mean*_ is mean velocity during this time interval, and *x(t)* is the time-dependent position. In our case, acceleration was the raw measured data. Therefore, we derived acceleration once to obtain the time-dependent jerk, and to obtain the time-dependent velocity we integrated acceleration once. The integration constant of the time-dependent velocity (*v*_*0*_) was set to zero because *t*_*1*_ was selected to match with an overall static position before the initiation of the shooting. High peak velocities indicate explosive movements, whereas low total jerk is related to smooth and continuous movements.

Additionally, we estimated the relative timing between the movements of the different limbs as the time lag with the maximal cross-correlation between two acceleration profiles. A coordinated set of time delays allows for a smoother transfer of energy from lower to upper and distal bodies, which facilitates accuracy in throwing tasks [39]. The center of pressure trajectory (CoP) was computed from the ground reaction forces and moments. The total variability with respect to the block average of the CoP in a trial in antero-posterior (AP) and medio-lateral (ML) axes was computed with the following expression:

$${CoP}_{VAR}^{i}=\frac{1}{∆t}\int_{t_{1}}^{t_{2}}{\left(\hat{CoP}-{CoP}_{i}\right)}^{2}dt$$ (2)

where $\hat{CoP}$ represents the average block trajectory of the *CoP* in either AP or ML directions, *CoP*_*i*_ represents the trajectory of the *CoP* during the *i-th* trial in the corresponding direction, and *Δt* is the time difference between *t*_*1*_ and *t*_*2*_. This measure of CoP variability was used to characterize movement stability for each free throw, as a convenient replacement for postural sway measures that can only be measured in completely stationary conditions [12].

### Data analysis

The results of cortisol levels, state anxiety, pleasure, arousal, dominance, performance scores (see S1 File) and movement execution were analyzed with repeated measures ANOVAs to reveal possible effects of Treatment (TSST, Placebo-TSST) and Time (with the levels that correspond to each variable). Overall level of significance was defined as *p* < .05. Effect sizes were calculated using partial eta-squared (*η*_*p*_²). Differences were evaluated post-hoc with Newman-Keuls tests. Statistical analyses were performed by Statistica 12 for PC. The neuroendocrine reaction was measured as the reactivity of salivary cortisol with respect to baseline.

A multivariate logistic model of scoring probabilities was used to determine the relative strength of the multiple constraints on shooting performance. The model attempts to predict the probability of scoring in a trial using all the variables measured in the experiment (i.e., physiological, psychological and kinematic variables). The fundamental assumption of this model is that each shot in a sequence is a statistically independent event and differences in scoring probability are due to the underlying psychological, physiological, and biomechanical processes. The independence of free throw sequences has been suggested by the studies that prove the inexistence of cold hand and hot hand effects in basketball [40]. In addition, the selected population was homogeneous with respect to basketball skillfulness and exposure. Thus, individual differences in scoring probability cannot be attributed to differences in expertise or style, indicating that shooting events between subjects can be also considered independent in our population. We proceed now to present the variables included in the model and the preprocessing required.

Cortisol and STAI values were linearly interpolated for each block of shots and participant to obtain a per-trial estimation. In addition, the first measure of cortisol for each participant and day was used as the baseline for the remaining measurements. The experimental factors Treatment (TSST, Placebo-TSST) and Time (pre-test, post-test) were also included in the regression as dummy variables. SAM tests were excluded from the regression as the analysis of the data revealed low variability values.

Variables were standardized to allow for direct comparison of the relative importance of each factor (the standardization did not change the results, only the relative scales of the estimated parameters). Standardized variables allow for direct and intuitive comparison of odds ratios: Odds ratios above 1 indicate an increase the scoring probability, whereas odds ratios below 1 indicate a decrease the scoring probability. For reasons of completeness we additionally report the non-standardized odds ratio for the independent variables with significant effects on the outcome. A χ^2^ test on the Log-likelihood ratio between the full model and the intercept-only null model was used to assess the goodness of fit of the model. Internal validation was performed using leave-one-out algorithm cross-validation. Wald’s test on the z-scores of the coefficients was used to assess which coefficients were significantly different from 0. Computation of the logistic model was performed using the R software [41] (see S2 and S3 Files).

## Results

### Treatment order

Repeated measures ANOVAs did not reveal significant interaction between factors Order (TSST-Placebo, Placebo-TSST), Treatment (TSST, Placebo) and Time (with the temporal levels that correspond to each measurement) on cortisol levels, state anxiety, pleasure, arousal, dominance, free throw performance and movement execution. Hence, the effects of treatment order can be disregarded in the present study [29].

### Salivary cortisol levels

**Fig 2:**
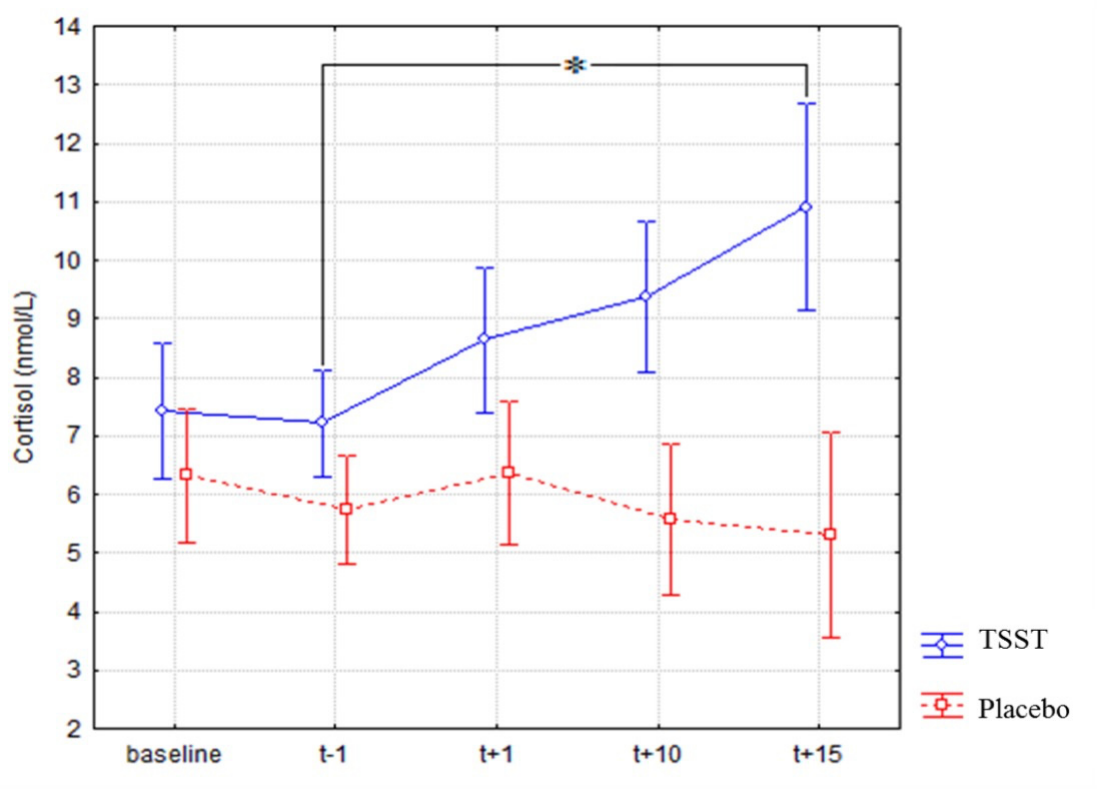
Salivary cortisol concentrations of participants exposed to the two treatments (TSST and Placebo-TSST). A significant increase of salivary cortisol was shown in the TSST treatment and no significant variation was shown in the Placebo-TSST treatment.

Results for salivary cortisol are presented in Fig 2. A repeated measures ANOVA with the factors Treatment and Time (baseline, *t-1*, *t+1*, *t+10*, *t+15*) revealed a significant effect of Treatment (*F*(1, 36) = 18.80, *p* = .000, *η*_*p*_² = .34) and Time (*F*(4, 144) = 3.25, *p* = .020, *η*_*p*_² = .08). There was a significant Treatment X Time interaction (*F*(4, 144) = 7.09, *p* = .000, *η*_*p*_² = .16). Newman-Keuls analyses evidenced that cortisol level increased significantly after the TSST between *t-1* (*M* = 7.21, *SD* = 2.08) and *t+15* (*M* = 10.91, *SD* = 4.88, *p* = .004). In contrast, there was no significant difference between these two measurement times after the Placebo-TSST. Moreover, a specific repeated measures ANOVA conducted on cortisol levels with the factors Treatment and Time (baseline, *t-1*) did not reveal any significant effect. This indicates that physical exertion involved in a sequence of 40 free throws do not influence salivary cortisol levels.

### State anxiety

**Fig 3:**
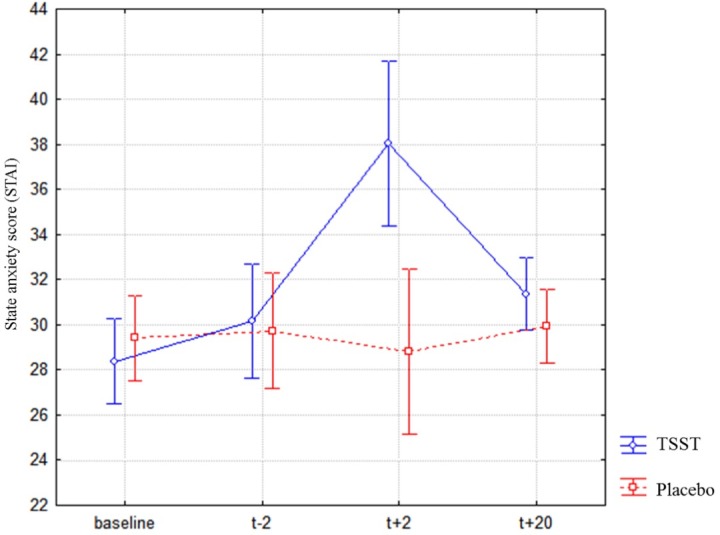
State anxiety of participants exposed to the two treatments (TSST and Placebo-TSST) measured with the STAI. A significant increase of state anxiety was shown after the TSST, but not after the Placebo-TSST. The measure was assessed just before or just after the saliva collection.

Results for state anxiety are presented in Fig 3. A repeated measures ANOVA with the factors Treatment and Time (baseline, *t-2*, *t+2*, *t+25*) revealed no significant effect of Treatment (*F*(1, 36) = 3.28, *p* = .078), a significant effect of Time (*F*(3, 108) = 8.98, *p* = .000, *η*_*p*_² = .19) and a significant Treatment X Time interaction (*F*(3, 108) = 12.77, *p* = .000, *η*_*p*_² = .26). Newman-Keuls tests showed that after the treatment (*t*+2) state anxiety was significantly higher after the TSST (*M* = 38.05, *SD* = 9.07) than after the Placebo-TSST (*M* = 28.78, *SD* = 6.39, *p* = .000). Consequently, the TSST resulted in higher anxiety ratings than the non-stress condition.

### Pleasure, arousal and dominance

**Fig 4:**
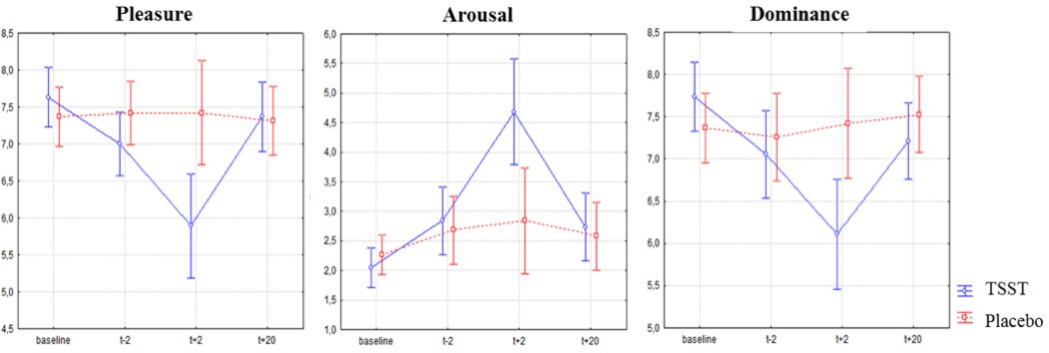
Pleasure, arousal and dominance of participants exposed to the two treatments (TSST and Placebo-TSST) measured with the SAM. A significant increase of arousal and a significant decrease of pleasure and dominance were shown after the TSST, whereas no significant variations were evidenced after the Placebo-TSST. These measures were assessed just before or just after the saliva collection.

Results for pleasure, arousal and dominance are presented in Fig 4. Three consecutive repeated measures ANOVAs were conducted on pleasure, arousal and dominance scores, with the factors Treatment and Time (baseline, *t-2*, *t+2*, *t+25*). They revealed that there was no significant effect of Treatment (*F*(1, 36) = 2.45, *p* = .126; F(1, 36) = 2.30, *p* = .138; and *F*(1, 36) = 1.76, *p* = .197, respectively), a significant effect of Time (*F*(3, 108) = 6.56, *p* = .000, *η*_*p*_² = .15; *F*(3, 108) = 15.51, *p* = .000, *η*_*p*_² = .30; and *F*(3, 108) = 6.79, *p* = .000, *η*_*p*_² = .15, respectively) and a significant Treatment X Time interaction (*F*(3, 108) = 7.81, *p* = .000, *η*_*p*_² = .17; *F*(3, 108) = 7.26, *p* = .000, *η*_*p*_² = .16; and *F*(3, 108) = 7.23, *p* = .000, *η*_*p*_² = .16, respectively). Newman-Keuls tests evidenced that pleasure and dominance were significantly lower after the TSST (*M* = 5.89, *SD* = 0.80 and *M* = 6.10, *SD* = 1.24 respectively) than after the Placebo-TSST (*M* = 7.00, *SD* = 0.84, *p* = .000 and *M* = 7.05, *SD* = 0.95, *p* = .000 respectively) whereas arousal was higher after the TSST (*M* = 4.68, *SD* = 1.94) than after the Placebo-TSST (*M* = 2.84, *SD* = 1.22, *p* = .000).

### Free throw performance

**Fig 5:**
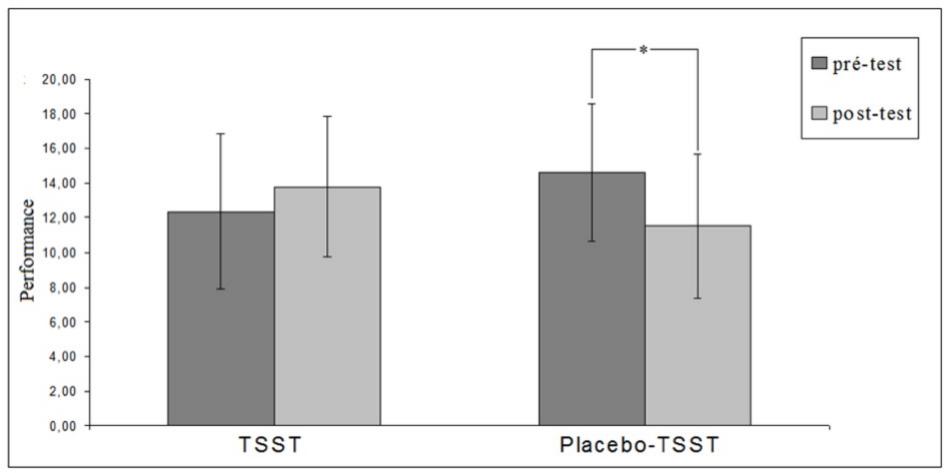
Number of successful free throws in pre-test (first sequence of 40 free throws, before the TSST or the Placebo-TSST) and post-test (second sequence of 40 free throws, after the TSST or the Placebo-TSST) depending on the treatment. A significant decrease of free throw performance was found after the Placebo-TSST, whereas no significant variation was evidenced after the TSST.

Results for free throw performance are presented in Fig 5. A repeated measures ANOVA with the factors Treatment and Time (pre-test, post-test) evidenced no significant main effect of Treatment or Time, but a significant interaction between these variables (*F*(1, 36) = 9.86, *p* = .003, *η*_*p*_² = .21). Newman-Keuls tests revealed that (a) in the pre-test there is no significant differences in free throws performance between the TSST (*M* = 12.36, *SD* = 4.44) and the Placebo-TSST (*M* = 14.63, *SD* = 4.05, *p* = .17), (b) when the participants were confronted to the Placebo-TSST, their free throw performance significantly decreased between the pre-test (*M* = 14.63, *SD* = 4.05) and the post-test (*M* = 11.52, *SD* = 4.15, *p* = .022) and (c) when the participants were confronted to the TSST, their free throw performance did not decrease and even increased but not significantly between the pre-test (*M* = 12.36, *SD* = 4.44) and the post-test (*M* = 13.79, *SD* = 3.99, *p* = .24). In sum, the Placebo-TSST treatment slightly decreased performance, whereas the TSST treatment had negligible improving effects on performance. To rule out the possible erratic behavior of a novice population, we have additionally tested pretest-posttest performance differences using a per-participant baseline. The results of these analyses fully replicate those of the repeated measures ANOVA and are thus not presented in detail.

### Free throw movement execution

The repeated measures ANOVAs of factors Treatment and Time (pre-test, post-test) on the movement kinematics, smoothness and stability yielded negligible effects. This indicates that neither the TSST nor the Placebo-TSST significantly influenced the execution of the shooting movements.

### Multivariate logistic model of scoring probabilities

The binary performance of each trial (scored vs. non-scored) was used as response variable of the logistic model, whereas STAI, cortisol level and movement execution measurements were used as independent variables. The distinct movement patterns of the limb and the trunk produced strong correlations on the kinematic measures from the same region of the body. To mitigate this multicollinearity, only the kinematic variables from sacrum and hand were included in the analysis. A total of 15 independent variables and 3013 observations were included in the analysis (with 986 scored trials in total) and 27 trials were discarded because at least one of the independent measures could not be measured. No additional variable selection was performed to prevent undue bias of the model.

Results for the multivariate logistic regression are summarized in Table 1. The model explains the scoring probabilities significantly better than the intercept-only null model (χ^2^ = 32.636, *p* = .002). The misclassification probability obtained by cross-validation was .22, in other words, the model had 78% of classification accuracy. Thus the model generalizes reasonably well and without excessive over-fitting. We found that scoring probability is significantly correlated with Peak Hand Speed, Total Sacrum Jerk and the time lags between Hand-Forearm and Arm-C7. All other variables did not significantly change the scoring probability. In particular, the experimental factors, the level of cortisol and the psychological state had no effect on scoring probabilities. This finding confirms the abovementioned lack of treatment effects on free throw performance. Interactions between variables were not significant. An increase of Total Sacrum Jerk (1% every arbitrary unit, with typical values of this variable lying in the interval 20 to 60) and Arm-C7 delay (2% every 10 ms) increased the scoring probability, whereas higher values of the Peak Hand Speed (56% every ms^-1^) and the Hand-Forearm delay decreased the scoring probability (5% every 10 ms).

**Table 1:** Results of the logistic regression.

	Beta		Odds Ratio			z-test	
	Mean	SEM	Mean	95% CI		z	P(>|z|)
**(Intercept)**	-0.729	0.039	0.48	0.45	0.52	-18.626	.000***
**Stress**	-0.015	0.045	0.98	0.90	1.08	-0.336	0.737
**Block**	-0.048	0.042	0.95	0.88	1.04	-1.138	0.255
**Cortisol**	0.009	0.043	1.01	0.93	1.10	0.197	0.843
**STAI**	-0.020	0.048	0.98	0.89	1.08	-0.407	0.684
**CoP ML**	-0.038	0.042	0.96	0.89	1.04	-0.911	0.362
**CoP AP**	-0.036	0.043	0.96	0.89	1.05	-0.829	0.407
**Speed Hand**	-0.166	0.057	0.85	0.76	0.95	-2.927	0.003**
**Speed Sacrum**	0.071	0.045	1.07	0.98	1.17	1.587	0.112
**Jerk Hand**	0.023	0.042	1.02	0.94	1.11	0.539	0.590
**Jerk Sacrum**	0.135	0.042	1.14	1.06	1.24	3.254	0.001**
**Timing H-F**	-0.087	0.041	0.92	0.84	0.99	-2.103	0.035*
**Timing F-A**	-0.038	0.043	0.96	0.88	1.05	-0.869	0.385
**Timing A-C7**	0.143	0.052	1.15	1.04	1.28	2.736	0.006**

From left to right, the columns include the mean value and standard error of the mean (SEM) of the exponential coefficients (*β*), the odds ratio mean value and a 95% CI, and Wald’s z-test with the null hypothesis that the coefficient is equal to zero. Abbreviations: CoP ML (per-trial variability of CoP displacement in the ML axis), CoP AP (per-trial variability of CoP displacement in the AP axis), H-F (Hand-Forearm), F-A (Forearm-Arm), A-C7 (Arm-C7).

*** *p* < 0.001

** *p* < 0.01

* *p* < 0.05.

## Discussion

The purpose of this research was two-fold. First, to assess the suitability of the TSST as a method to induce stress on performance in realistic sport contexts. The assessment of the TSST was indeed successful. The neuroendocrine and psychometric data suggest that the TSST was stressful for the participants while the Placebo-TSST had no effect. Consequently, the post-test sequence of free throws after the TSST was executed in an effective state of stress. The second aim of the study was to analyze the execution and performance of basketball free throws in neutral and psychosocially stressful contexts. We found no effect of the TSST on overall shooting performance and a mild decrease of performance after the Placebo-TSST. With respect to the control of movements, analysis of average group behavior evidenced no systematic effect of neither the TSST nor the Placebo-TSST. A multivariate regression model that considers performance on a trial-by-trial basis evidenced that timing, smoothness and explosiveness of the movements are relevant factors to improve performance. The per-trial analysis confirmed that TSST-induced stress, cortisol levels and self-reported anxiety had no effect on beginners’ shooting performance. In the following paragraphs we will take a closer look at these findings and discuss their importance within a sport-specific context.

### TSST assessment in a sport context

A significant rise in salivary cortisol and anxiety levels was found after the stress induction, in agreement with the literature focused on the biological and psychological markers of stress following the TSST (for a review, seen [7]). In contrast, there was no increase of cortisol and anxiety in response to the Placebo-TSST. Since this second condition was non-stressful it could serve as a perfect control condition [27]. Moreover, it should be noted that the present study provides additional information to facilitate the application of the TSST in sport contexts. For example, no effect of treatment order on cortisol levels and state anxiety was found, in line with the findings related to the Placebo-TSST [27]. In addition, and following studies in other sport contexts (e.g., [9,26] with tennis serve), salivary cortisol levels were not influenced by the physical exertion in a sequence of 40 free throws: salivary cortisol did not increase during the pre-test sequence of free throws under the two conditions and during the post-test sequence in the Placebo-TSST condition. Consequently, this sport-related setting makes it possible to clearly link the rise in cortisol levels to the psychological factor.

Different aspects of emotional experience (pleasure, arousal and dominance) were assessed with the Self-Assessment Manikin [11], a non-verbal pictorial assessment technique. SAM has been scarcely used in the TSST literature [42]. However, it is an easy method for quickly assessing reports of affective response generated by the TSST, which would be useful when repeated measures are necessary as in the TSST protocol. Moreover, identifying the dominance emotional state (i.e. the lack of control) is particularly interesting, because one of the effective components of the TSST is the uncontrollability of the stressor tasks. In the present study, pleasure and dominance were lower after the TSST than after the Placebo-TSST and arousal was higher. Used with other subjective measures (such as anxiety or mood, see [7]) and physiological ones such as cortisol, emotional states assessed with the SAM may be useful to complete the identification of the subjectively negative experience induced by the TSST.

### Performance of basketball free throw

Performance significantly decreased after the Placebo-TSST condition and remained stable after the TSST condition. These findings may appear surprising because the psychosocial stress induced by the TSST is considered as particularly acute [7,25]. Since free throwing is a psychological demanding activity as such, we expected that the additional acute stress induced by the TSST treatment would have a negative or even detrimental effect on free throw performance. As this was not the case, the present findings are difficult to interpret as resulting from an inverted U-shape relationship between stress and free throw performance for novices in basketball. Therefore, rather than oversimplifying the complex phenomena related to stress using a general U-shape law, we consider stress as a General Adaptation Syndrome [43]. From this perspective, individual differences in stress perception and response can exist without any measurable neuroendocrine difference [44]. In this sense, stress may have positive or negative impacts on performance depending on factors as disparate as the context, the motivational and physiological state or the personal biography. Moreover, the “distress-eustress” model [45] attempts to distinguish between “bad stress” and “good stress” using the notion of adaptation to the perceived environment. The “good stress” is a process leading to positive adaptation (or allostasis, a better coupling with the perceived environment) [46]. For example, the stress induced by the TSST improves spatial memory for map routes [47], with an increase in the automatic information processing. On the contrary, the "bad stress" is a process leading to negative adaptations to the environment. For instance, acute stress generated by the TSST reduces speech fluency [48]. These findings suggest that stress should not be regarded as a psychological factor that is linked to performance in a simple straightforward manner. In contrast it is a very complex anticipatory adaptation to the environment leading to compensating reactions that may be positive or negative depending on a number of contextual variables. In this sense, the stability of free throw performance following the TSST indicates that the stress created by the TSST was used as a “good stress” by the novice participants, which could have prevented the decline of their free throw performance present in the placebo-TSST condition.

The fact that anxiety is always detrimental to sport performance should be considered as a “myth” [49]. Indeed, the study of the influence of anxiety on sport performance is very widespread in sport psychology and most studies have tried to provide explanation for “choking under pressure” (i.e. decrease of performance under stressful conditions [50]). However, the performance of many athletes did not decrease under stress, and even increased in some cases [51]. Performance can be affected by anxiety and physiological arousal, and these influences can be either positive or negative [52]. According to the directional perspective [53] anxiety symptoms may be interpreted by the player as hampering sport performance, they may also be interpreted as facilitative. In the present study, the resulting adaptation to the stressor may have led the participants of the present study to maintain their free throw performance.

### Integrated analysis of basketball free throw under stress

We found no effect of stress on any of the parameters of free throw execution considered: smoothness, explosiveness, temporal coordination and dynamical stability. The low reliability of these measures in a novice population is very common due to the strongly varying styles, and the lack of consistency in the execution of the technique [54,55]. Using group averaging with a novice population can be misleading, because intra-individual variability is comparable to inter-group variability. Therefore, we used a multivariate logistic regression model that can account for the relative influence of the different factors that affect the probability of scoring a free throw shot. This model used all the relevant variables of our design, including the experimental factors, cortisol levels, psychological state and kinematic and dynamic parameters of movements. The model confirmed the absence of effects of TSST-induced stress and its related physiological and psychological responses (cortisol levels and psychological states) on shooting performance.

Indeed, the model evidenced interesting relationships between movement control and free throw shooting performance. We found that smoothness (total jerk) in the pelvic movements decreased performance. This effect maybe reflecting the different shooting styles in novices: some players shoot with the lower body and the trunk practically static, while others use more extensive body movements to shoot. These opposing styles can explain the apparent contradiction that less trunk smoothness is beneficial for shooting. We also found that an excessively explosive movement of the shooting hand decreases performance. Wrist flexion during shooting must be explosive, but an excess of momentum complicates the fine-grained control required for long distance precision throwing [55]. Finally, two temporal relationships between acceleration profiles were found to be the most relevant factors to increase shooting performance: trunk-arm delay and hand-forearm delay. In general, expert performance in precision throwing tasks goes together with a positive time delay from lower to upper and distal bodies of the kinematic chain [39,55]. This full body coordinated movement allows for an optimal transfer of energy from lower body up to the hand, so that the final joint of the chain does not need to generate high energy/power, but to finely control the final release movement [41]. For the specific case of basketball, the delay between wrist and elbow flexion is the only negative delay in the chain of power transmission: the peak wrist acceleration appears (slightly) earlier than the peak elbow acceleration in expert players [56]. In our analysis, we found that a lower (i.e., more negative) hand-forearm delay and a higher arm-trunk delay increase performance. This confirms the relevance of appropriate energy transmission across the segments of the kinematic chain for successful shooting.

In sum, the wildly varying styles and the low consistency in the technical execution of novices make it difficult to evaluate the effect of stress on movement execution. Future studies should be conducted with elite or professional basketball players because they produce very stable and stereotyped movement patterns [56]. Moreover, even if elite sportsmen displayed lower cortisol and psychological responses to the TSST than untrained men [29,30], their mean anxiety was greater for high-level athletes than lower-level athletes [17]. Consequently, variations in the control of free throw might be more easily highlighted between the TSST and the Placebo-TSST conditions.

## Conclusion

To the best of our knowledge, this study is the first to highlight the influence of the complete TSST on a specific sport skill, namely the free throw in basketball, as compared to the Placebo-TSST condition. The data showed a differentiated influence on free throw performance between stressful and non-stressful conditions: performance decreased with the Placebo-TSST treatment, whereas it remained stable with the TSST. Moreover, because participants were novices in basketball, data relative to the movement control of the free throw movement did not evidence any influence of stress. Nevertheless, we found some interesting effects of movement control and execution on shooting performance. Timing, smoothness and explosiveness of the movements are more relevant to account for beginner’s behavior than physiological and psychological states. Future research should extend the use of the TSST in sport contexts with different levels of expertise, different sports and different motor skills, and with an interdisciplinary approach to understand the underlying processes.

## Supporting Information

S1 File Synthetesis of the data (cortisol levels, free throw performance, STAI scores, SAM scores) (XLS)

S2 File R script that performs the logistic model. (CSV)

S3 File Dataset used in the logistic model. (R)
